# Social Learning in Insects: A Higher-Order Capacity?

**DOI:** 10.3389/fnbeh.2012.00057

**Published:** 2012-09-05

**Authors:** Martin Giurfa

**Affiliations:** ^1^Centre de Recherches sur la Cognition Animale, Université de ToulouseToulouse, France; ^2^Centre de Recherches sur la Cognition Animale, Centre National de la Recherche ScientifiqueToulouse, France

Insects possess miniature brains but exhibit a sophisticated behavioral repertoire (Menzel and Giurfa, 2001; Giurfa, 2003, 2007; Chittka and Niven, 2009; Srinivasan, 2010; Avarguès-Weber et al., 2011; Dyer, 2012; Zhang et al., 2012). Recent studies have indicated that insects copy the behavior of conspecifics in contexts as diverse as foraging, in the case of bumblebees (Leadbeater and Chittka, 2005, 2008; Worden and Papaj, 2005), mate choice in the case of flies (Mery et al., 2009), and predator avoidance in the case of crickets (Coolen et al., 2005). These reports yield new light on the cognitive richness of insect behavior, which seems to transcend basic Pavlovian and operant learning, and have received a wide coverage, thereby inducing a reappraisal of insect learning capabilities. Yet, the critical question is not whether or not insects achieve “marvelous feats,” but, essentially, how do they achieve them. Here I focus on recent studies on social learning in insects and analyze to what extent these learning cases exceed elemental-learning interpretations, i.e., interpretations based on simple stimulus–stimulus (Pavlovian) or behavior-stimulus (operant) associations.

**Figure 1:**
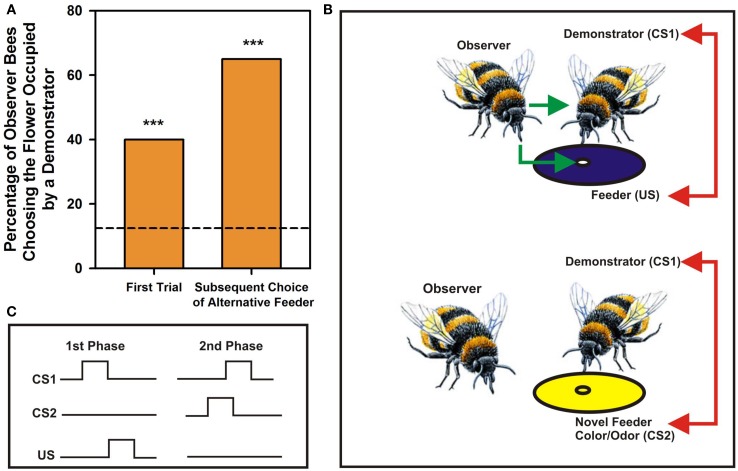
**Social learning in bumblebees – an elemental account**. **(A)** Percentage of choices by observer bees of a feeder occupied by a demonstrator bee. The arena contained eight feeders, four blue and four yellow. The demonstrator was placed on one feeder type, yellow or blue, and the observer released in the arena. Right bar: Choices of the feeder occupied by a demonstrator in the first trial, when both feeder types were unfamiliar to observers. Left bar: Choices of the alternative feeder type in subsequent trials when it was occupied by a demonstrator. The dashed line corresponds to a random choice in a situation where eight feeders were available. Asterisks correspond to *p* < 0.01. Adapted from Leadbeater and Chittka (2005). **(B)** Possible associations established by bumblebees during social learning in a foraging context. During direct interactions with demonstrators, observers experience nectar reward (US; green arrow) and associate demonstrators (conditioned stimulus 1 or CS1) with the US (red arrow); if demonstrators come to choose a novel feeder (here with a different color), observers will also land on the novel occupied feeder and will associate the physical properties of the flowers that demonstrators now exploit (CS2) with the demonstrators themselves (CS1; red arrow). The process postulated corresponds to a case of second order conditioning. **(C)** Nature of associations established during the two phases of a second order conditioning process.

Bumblebees learn foraging preferences from other bumblebees by observing their choices of visual rewarded targets. They land on unknown flowers if other bees (the demonstrators) are already present on them (Leadbeater and Chittka, 2005, 2009; Figure 1A, left column). If these demonstrators are present on a different flower morph (i.e., displaying a different color; see Figure 1B), observers will also tend to land on the novel morph despite having experienced a different color on their first choice (Figure 1A, right column). Observation of conspecifics choices without direct interaction may also determine own actions: when naïve bees are separated from experienced foragers by a transparent screen such that they can observe experienced foragers choosing artificial flowers but can neither sample the flowers by themselves nor interact with their foraging conspecifics, they choose afterward the flowers preferred by the experienced demonstrators (Worden and Papaj, 2005). Similarly, naïve bumblebees abandon an unrewarding flower species and switch to a more rewarding alternative more quickly when accompanied by experienced foragers (Leadbeater and Chittka, 2007).

Wood crickets *Nemobius sylvestris* also exhibit social learning as they learn to hide under leaves from experienced conspecifics in the presence of a natural predator, the wolf spider (*Pardosa* sp.; Coolen et al., 2005). Observer crickets were placed in a leaf-filled arena accompanied by conspecifics that were either confronted with a wolf spider and tended, therefore, to hide under leaves, or that did not experience this predatory threat. Observers that interacted with spider-experienced conspecifics were more likely to hide under leaves than observers that interacted with demonstrators which had no recent spider experience. This difference persisted 24 h after demonstrators were removed from the experimental arena, thus showing that perception of danger in observers had been altered by the demonstrators’ behavior (Coolen et al., 2005). Interestingly, crickets did not hide under leaves when separated from demonstrators by a partition that allowed for pheromone exchange between compartments but not for visual or physical contact; nor did they increase their tendency to hide when placed in arenas that had previously contained crickets confronted with spiders. Thus, naïve crickets learned from experienced demonstrators how to hide under leaves when facing a potential threat and this learning required a direct contact between observers and demonstrators.

Another example of learning in a social context is provided by the fruit fly in a mating and an oviposition context (Mery et al., 2009; Battesti et al., 2012). Two male phenotypes were artificially generated by dusting individuals with green or pink powder. In this way, females could differentiate between these two types of male (Mery et al., 2009). An observer female was placed in a glass tube from which it could see the interaction between a painted male and another female. In one case, the male which was, say green, copulated with the demonstrator female, and in other case, the other male which was, say pink, did not copulate because it was paired with a non-receptive female. After this double demonstration, the observer female was presented with two new males, one pink and the other green. Observer females preferably mated with males of the color that was associated with a successful copulation, over males of the color which were associated with unsuccessful copulative attempts. This effect disappeared when observer females were impeded to observe directly the other flies during their interaction (Mery et al., 2009). Comparable results were found in an oviposition context (Battesti et al., 2012); the choice of oviposition sites by female fruit flies is strongly influenced by experienced demonstrator females, which have been conditioned to avoid one of two equally rewarding media that presented two different scents (strawberry vs. banana). Naïve observer flies develop a preference for the same medium that experienced demonstrator flies learned to choose, even if for observers the alternative medium would be equivalent. Such oviposition site preference was socially transmitted from demonstrators to observers even when they interacted in a cage with only unflavored, pure agar medium, and even when the observer flies had previous personal experience with both rewarding media (Battesti et al., 2012).

Observing conspecifics and then deciding about own actions are, therefore, capabilities that are also present in miniature brains. Yet, as fascinating as they may appear, none of these works provide insights into the mechanisms responsible for these behaviors. A fundamental exercise is, therefore, to determine whether elemental accounts, based on simple associative links, can explain the insect behavior in these different contexts. This seems plausible in most, if not all, cases considered. Transmission of oviposition site preference, for instance, could be a case of simple appetitive olfactory learning as already studied in the fly (Colomb et al., 2009). Demonstrator flies were trained to choose a rewarding media impregnated with strawberry odor. This odorant can therefore adhere to the cuticular surface of demonstrator flies and act as conditioned stimulus (CS) for observers in direct contact with them. The unconditioned stimulus (US) acting in this context is more difficult to define, but recent work has shown that mating alters dramatically cuticular hydrocarbon profiles in a sex-specific manner in flies (Everaerts et al., 2010). If oviposition induces a similar effect, some of the compounds detectable on the cuticular surface of ovipositing females could act as positive indicators of oviposition success and thus, as biologically relevant reinforcements to be associated with the strawberry odor. The whole process would constitute a simple case of classical conditioning in which observer flies learn to associate strawberry odor with an oviposition signal, both present on the cuticula of demonstrators. Later, when confronted with two equally rewarding media, one scented with strawberry and the other with banana, observers choose preferentially the strawberry one based on simple associative learning.

The case of bumblebee social learning in a foraging context, described above (Leadbeater and Chittka, 2005), can also be interpreted in terms of an elemental form of associative learning called second-order conditioning (Pavlov, 1927), which involves two connected associations. In second-order conditioning, an animal first learns an association between a CS and an US and then experiences a pairing between a new CS2 and CS1 so that CS2 becomes a predictor of CS1, and indirectly of the US (Figure 1C). In this scenario, the observer bee would first learn through joint foraging activities an association between a demonstrator bee, acting as CS1, and nectar reward, acting as US. Observation of the foraging choices by the demonstrator may then lead the observer bee to establish a novel association, this time between a novel color and/or odor chosen by the demonstrator, acting as CS2, and the demonstrator itself, the CS1. In this way, the novel color/odor becomes meaningful through its association with CS1, and indirectly with the US (Leadbeater and Chittka, 2007; Figure 1B). This hypothesis is supported by the fact that honey bees and fruit flies can learn such second order associations. While flies exhibit second order conditioning in an aversive context, in which they learn to associate an odor (CS1) with shock (US) and then a second odor (CS2) with the previously conditioned CS1 (Tabone and de Belle, 2011), honey bees learn second order associations in an appetitive context while searching for food. They learn to connect both two odors (Odor 1 + Sucrose Reward; Odor 2 + Odor 1; Takeda, 1961; Bitterman et al., 1983; Hussaini et al., 2007) and one odor and one color (Grossmann, 1971), thus rendering the second order conditioning explanation of social learning plausible.

Further explanations could be provided for other cases of insect social learning which should be analyzed as cases of individual learning of social cues. As such, very simple mechanisms based on elemental associations, either Pavlovian or operant, may account for these phenomena. From this perspective, social learning in animals with miniature brains should not be considered as a surprising or highly cognitive ability given that it would be based on simple associative learning, which has been intensively studied in these animals (Menzel, 1999; Heisenberg et al., 2001; Daly et al., 2004; Mizunami et al., 2004, 2009; Davis, 2005; Dupuy et al., 2006; Giurfa, 2007; Keene and Waddell, 2007; Busto et al., 2010; Giurfa and Sandoz, 2012). Thus, rather than highlighting the fact that insects are capable of social learning, researchers should put the accent on the identification of the cues that are associated and on the analysis of their processing in the insect brain.
